# Single-trocar thoracoscopic pericardio-pleural fenestration under local anesthesia for malignant pleural effusion: a case report

**DOI:** 10.1186/s40792-019-0694-6

**Published:** 2019-08-27

**Authors:** Masatsugu Ohuchi, Shuhei Inoue, Yoshitomo Ozaki, Yuki Namura, Keiko Ueda

**Affiliations:** 1 Department of General Thoracic Surgery, National Hospital Organization Higashi-Ohmi General Medical Center, 255 Gochi-cho, Higashi-Ohmi, Shiga 527-8505 Japan; 2 Department of Thoracic Surgery, Rakuwakai Otowa Hospital, Kyoto, Japan

**Keywords:** Thoracoscopy, Local anesthesia, Pericarditis, Lung cancer, Pleural biopsy

## Abstract

**Background:**

Pericardio-pleural fenestration by video-assisted thoracoscopic surgery is an efficient procedure for malignant pericardial effusion, but requires general anesthesia with single-lung ventilation.

**Case presentation:**

A 43-year-old woman was referred with complaints of deteriorating dyspnea and orthopnea. Chest computed tomography revealed right massive pleural effusion and pericardial effusion. Echocardiography demonstrated collapse of both the right atrium and right ventricle due to cardiac tamponade. Semi-rigid thoracoscopic pleural biopsy and pericardio-pleural fenestration were successfully performed under local anesthesia via a single trocar, because surgical procedures under general anesthesia with single-lung ventilation might have been intolerable for the patient. Adequate biopsy specimens of pleura and pericardium and immediate relief of serious symptoms were obtained without perioperative complications. No recurrence of pleural or pericardial effusion was observed for 3 months postoperatively.

**Conclusion:**

Thoracoscopic pericardio-pleural fenestration under local anesthesia via a single trocar is feasible as an alternative approach in critically ill patients, allowing effective pericardial drainage, evaluation of the pleural cavity, and accurate biopsies of the pericardium and parietal pleura simultaneously.

## Background

Pericardio-pleural fenestration by video-assisted thoracoscopic surgery (VATS) is an efficient procedure for malignant pericardial effusion [1], but requires general anesthesia with single-lung ventilation.

This report describes semi-rigid thoracoscopic pericardio-pleural fenestration and pleural biopsy simultaneously via a single trocar under local anesthesia for a patient with malignant pericardial and pleural effusion.

## Case presentation

**Fig. 1:**
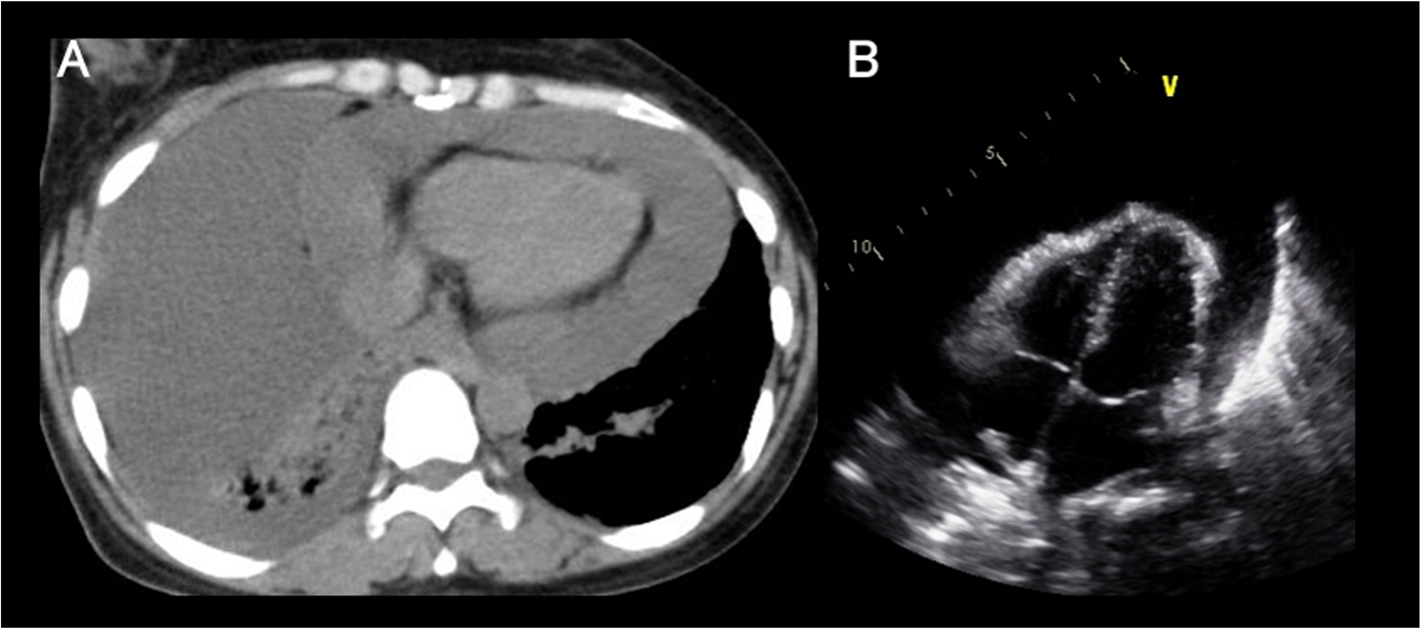
Radiologic and echocardiographic findings. **a** Chest computed tomography reveals right massive pleural effusion and pericardial effusion. **b** Echocardiography reveals massive pericardial effusion with right ventricle collapse

A 43-year-old woman was referred to our department complaining of dyspnea and orthopnea deteriorating over the course of about 2 weeks. She had a history of colon cancer 8 years earlier and had been found to have a lung tumor in the lower lobe of the right lung on chest computed tomography (CT) 7 years earlier. She had declined further examination and treatment for the lung nodule despite growth of the nodule. Body temperature was 36.6 °C, blood pressure was 114/85 mmHg, heart rate was 84 beats/min, and SpO_2_ was 96%. Stridor was heard and respiratory sounds were decreased in the right lung. Chest CT revealed right massive pleural effusion and pericardial effusion (Fig. 1a). Echocardiography demonstrated normal left ventricular systolic function but collapse of both the right atrium and right ventricle (Fig. 1b). She is difficult to take not only supine position but also Trendelenburg position due to massive pleural effusion and pericardial effusion; therefore, pericardiocentesis was impossible without sedative agents. Furthermore, she had declined further treatment as well as invasive examinations on the admission. Because hemodynamics was not broken yet and development of re-expansion pulmonary edema and circulatory failure due to positive pressure ventilation and sedative agents were concerned, we decided to perform elective thoracoscopy under local anesthesia and removal of pleural effusion and subsequently pericardial effusion, after her consent for treatments and the symptoms were relieved by thoracentesis.

**Fig. 2:**
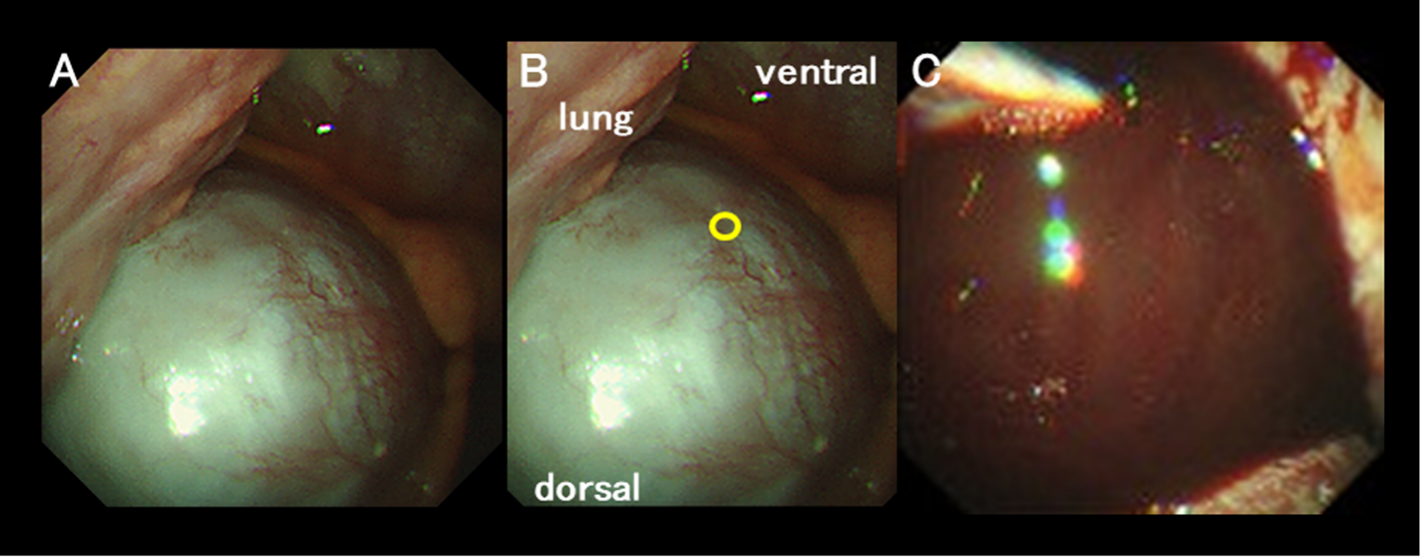
Thoracoscopic findings. **a** Thoracoscopic examination reveals many pleural nodules and severely distended pericardium with pericardial effusion. **b** A tiny pericardial window, about 5 mm in diameter, was created using biopsy forceps (circle). **c** Bloody pericardial effusion spurts through the pericardial fenestration to the right thoracic cavity

The pleural effusion (about 500 ml) was removed by thoracentesis, and a few atypical cells were detected. Because surgical procedures under general anesthesia with single-lung ventilation might have proven intolerable, thoracoscopic examination and pleural biopsy were performed under local anesthesia and light sedation by midazolam. The patient was positioned in a left decubitus position, and a 7-mm trocar was inserted into the right thoracic cavity. A single-channel thoracoscope (Olympus LTF-260™; Olympus, Tokyo, Japan) was inserted through the trocar. Examination revealed 800 ml of serous pleural effusion, many pleural nodules, and severely distended pericardium with pericardial effusion (Fig. 2a). The distended pericardium was broken by biopsy forceps at the ventral part of the phrenic nerve (Fig. 2b), followed by the evacuation of the pleural effusion and biopsies of the parietal pleura. About 435 ml of bloody pericardial effusion spurted through the pericardial fenestration (Fig. 2c). Finally, a 20-Fr double-lumen chest tube was inserted. The entire procedure took 35 min. Atypical cells were also detected in the pericardial effusion, and the histopathological diagnosis was primary pulmonary adenocarcinoma with neither EGFR nor ALK mutations. On postoperative day 10, talc pleurodesis was performed, and the chest tube was removed on day 14. Although 2 cycles of systemic chemotherapy with carboplatin, pemetrexed, and bevacizumab were performed and achieved stable disease, she refused to continue chemotherapy. No recurrence of pleural or pericardial effusion was observed for 3 months, at which time she transferred from the hospital into palliative care.

## Discussion

Drainage of pericardial effusion is recommended for symptomatic patients with a large volume of pericardial effusion. This palliative treatment often results in relief from symptoms and prolongation of survival following anti-cancer therapy, including systemic chemotherapy. Various approaches have been described for the diagnostic and therapeutic assessment of pericardial effusion, including pericardiocentesis, percutaneous catheter drainage, subxiphoid pericardial fenestration, and pericardial fenestration through thoracotomy or VATS. Pericardiocentesis and percutaneous catheter drainage are convenient methods for immediate relief of symptoms, but carry a risk of complications such as cardiac injury, arrhythmia, and cardiac arrest. Furthermore, about 40% of pericardial effusions recur after these methods [2]. Subxiphoid pericardial fenestration is also a widely accepted technique, but only allows for limited pericardial resection because of the restricted access with poor visual field and the inability to obtain information on the hemi-thoracic cavity. Pericardio-pleural fenestration by VATS under general anesthesia is a safe, less-invasive technique that allows not only pericardial drainage, but also collection of pleural effusion and pleural biopsy specimens [1]. However, general anesthesia with single-lung ventilation is often not intolerable for patients with pericardial effusion, which develops in some cases as a near-terminal event.

In recent years, many authors have reported that semi-rigid thoracoscopy under local anesthesia is useful as a diagnostic and therapeutic tool for various pleural diseases [3–5].

In the present case, a single-trocar thoracoscopic pericardio-pleural fenestration was successfully performed at a suitable operative time under local anesthesia and light sedation. Hemostasis of both the pleura and pericardium were achieved with no intraoperative complications. Adequate biopsy specimens of pleura and pericardium and immediate relief from serious symptoms were obtained.

Furst and colleagues proposed that, in patients presenting with both cardiac tamponade and massive pleural effusion, drainage of the pleural effusion should be given priority because sudden evacuation of the pericardial effusion leads to acute hemodynamic instability caused by failure of the right ventricle and decreased pulmonary circulation due to the large pleural effusion compressing the lung [6]. Furthermore, they described spontaneous respiration as essential to preserve the unstable hemodynamic state of the patient.

Katlic and Park have reported thoracoscopic pericardio-pleural fenestration under local anesthesia [4, 7]. Katlic and colleagues performed the procedure using camera, grasper, and scalpel through via two or three ports. In terms of single port, our method is similar to the one of Park and colleagues. They created pericardio-pleural fenestration after incision of pericardium using diathermy and added biopsy. We inserted biopsy forceps via a channel of semi-rigid thoracoscopy and created a tiny window. Because our procedure was operated via the channel of semi-rigid thoracoscope, the operation performance of forceps was easy. However, the available instruments are limited to the size of the forceps that can insert through the channel of thoracoscope.

Regarding one problem with this technique, Piehler and colleagues suggested that a direct relationship existed between the extent of pericardium resection and the incidence of recurrence or development of constriction [8]. Although only a tiny pericardial window, about 5 mm in diameter, was created using biopsy forceps in this case, no recurrence of pericardial effusion has been observed within the short period of follow-up. One of the reasons of no recurrence might be early induction of effective chemotherapy because of the minimally invasive procedure.

## Conclusion

A single-trocar thoracoscopic pericardio-pleural fenestration under local anesthesia is a feasible approach in critically ill patients that allows effective pericardial drainage, evaluation of pleural cavity, and accurate biopsy of pericardium and parietal pleura simultaneously. Rapid postoperative recovery can lead to early induction of systemic anti-cancer therapy.

## Data Availability

The datasets supporting the conclusions of this article are available in the repository.

## Abbreviations

CT: Computed tomography
VATS: Video-assisted thoracoscopic surgery
